# Left ventricular dysfunction in sickle cell disease: the value of an electrocardiographic marker of increased risk of arrhythmia

**Published:** 2011-04

**Authors:** I Oguanobi

**Affiliations:** Department of Medicine, Federal Medical Centre, Asaba, Delta State, Nigeria

Sickle cell disease is one of the most prevalent genetic diseases worldwide; affecting 1/400 individuals of African descent as well as people of Arab, Indian and Hispanic descents.^1^-^3^ Abnormalities of cardiovascular function have increasingly been documented in sickle cell disease patients. Reports from several clinical studies in recent times have drawn attention to some ‘emerging’ cardiac pathologies in sickle cell disease and their potentially negative impact on cardiovascular function in these patients. Among these include myocardial infarction without coronary artery disease, pulmonary hypertension and cor pulmonale.^4^-^7^ Moreover, sudden unexpected death has become increasingly recognised as an important clinical feature of both the homozygous and heterozygous sickling syndromes; although the exact nature and its cause has remained unexplained.^8^-^10^

The emergence of cardiac complications in sickle cell disease patients could be attributed to the increasing life expectancy observed in these patients. Recent data indicates that 86 to 90% of patients survive to beyond 20 years of age.^11^ With the continued development of improved management and supportive care for patients with sickle cell anaemia and the resultant increase in life span, the spectrum of cardiac dysfunction is likely to enlarge in the future.

The mechanism underlying cardiac dysfunction in sickle cell anaemia has been extensively studied and multiple mechanisms have been proposed. In addition to the impaired microvascular circulation from intravascular plugs of sickled erythrocytes, other contributory factors include: extensive fibromuscular dysplastic narrowing of small cardiac arteries, non-inflammatory focal degeneration and apoptosis, platelet abnormalities or similar stimuli for endothelial and smooth muscle proliferation.^12^-^14^ The hyperkinetic circulation as a result of chronic anaemia contributes to eccentric ventricular hypertrophy and cardiomegaly, and the severity of cardiac chamber dilatation progresses with increasing anaemia.^5^,^15^ Despite myocardial remodelling/hypertrophy, the patients have increased myocardial wall stress as well as impaired ventricular relaxation.^16^

Data from clinical studies evaluating left ventricular systolic function using load-independent measures of myocardial contractility have revealed significant systolic dysfunction in sickle cell anaemia patients.^17^,^18^ The development of left ventricular systolic and/or diastolic dysfunction in sickle cell anaemia is associated with increased morbidity and mortality.^19^ There is a large body of evidence showing that diastolic dysfunction in sickle cell disease contributes to pulmonary hypertension and represents an independent predictor of mortality in these patients.^19^

It has been recognised that ischaemic phenomena associated with sickle cell anaemia could elicit morphological and functional abnormalities in the cardiac conducting system, resulting in paroxysmal arrhythmia and could further worsen the ventricular dysfunction.^5^ Such electrical instability induced by myocardial ischaemia has been postulated to be the cause of sudden cardiac death in patients with sickle cell disease.^5^,^10^

In the presence of left ventricular diastolic dysfunction, atrial fibrillation and indeed any form of arrhythmia causes significant cardiac decompensation. Atrial fibrillation in sickle cell disease is believed to be due to increase in atrial size with accompanying advanced atrial remodelling and profound global electrophysiological changes in refractoriness. Additional factors affecting atrial refractoriness include autonomic impairment, scars, and changes in the cellular membrane function.^20^ Several non-invasive electrocardiographic indicators have been investigated to predict the occurrence of arrhythmia in left ventricular diastolic dysfunction. On a 12-lead surface electrocardiogram, P-wave dispersion, because of its relationship to the non-homogenous and interrupted conduction of sinus impulses both intra- and interatrially, is recognised as a non-invasive marker of risk of atrial fibrillation.^21^

In the light of this, one pertinent question needs to be addressed: what is the clinical utility of P-wave dispersion in sickle cell anaemia? A step towards unravelling this puzzle would involve the examination of the relationship between P-wave dispersion and measures of left ventricular function in sickle cell anaemia patients, and the comparison of the indices with those of appropriately matched controls. In this connection, the article in this issue, ‘P-wave dispersion: relationship to left ventricular function in sickle cell anaemia’ is of relevance. The authors showed that P-wave duration and P-wave dispersion were significantly increased in sickle cell anaemia and that P-wave dispersion had a negative correlation with indices of left ventricular diastolic function. This novel study provides an interesting insight into the potential value of this simple electrocardiographic tool in the evaluation of ventricular function in sickle cell anaemia. This is especially useful in resource-limited areas of developing countries where access to modern investigative modalities is lacking. Major challenges in the use of this tool are the difficulty in standardisation of methods and the lack of acceptable normal limits of P-wave dispersion in the general population.

It is expected that this pilot study will stimulate further research efforts to determine the diagnostic/normal cut-off values, and specificity and sensitivity, as well as the long-term prognostic significance of increased P-wave dispersion in sickle cell disease.
